# Effects of probiotic supplementation on intestinal flora, brain–gut peptides and clinical outcomes in children with anorexia nervosa

**DOI:** 10.1017/S0007114525000169

**Published:** 2025-02-28

**Authors:** Xiaoyan Lu, Yali Liu, Longxia Hao, Junqiong Li, Linjuan Hua

**Affiliations:** 1 Department of Children’s Rehabilitation, The Eighth Hospital of Shijiazhuang, Shijiazhuang 050000, People’s Republic of China; 2 Department of Pediatrics, Xingtang County People’s Hospital, Shijiazhuang 050600, People’s Republic of China; 3 Department of Cardiology, Xingtang Hospital of Traditional Chinese Medicine, Shijiazhuang 050600, People’s Republic of China; 4 Department of Surgery, Xingtang Hospital of Traditional Chinese Medicine, Shijiazhuang 050600, People’s Republic of China

**Keywords:** Anorexia nervosa, Probiotics, Intestinal flora, Brain–gut peptides, Children

## Abstract

This study aims to assess the therapeutic effects of probiotic oral therapy in paediatric patients with anorexia nervosa (AN) and to investigate its impact on intestinal flora composition, brain–gut peptide levels and overall clinical outcomes. A retrospective study was conducted involving 100 children diagnosed with AN at Xingtang County People’s Hospital between January 2023 and June 2024. Patients were divided into two groups: a control group (*n* 50) receiving zinc gluconate oral solution alone and an observation group (*n* 50) receiving zinc gluconate plus probiotics. Outcome measures included intestinal flora analysis, brain–gut peptide levels (somatostatin (SS) and nitric oxide (NO)), clinical efficacy, serum trace element levels (Ca, Zn and Fe) and prognosis, including recurrence rates 6 months post-treatment. Baseline characteristics were similar between the two groups (*P* > 0·05). After treatment, the observation group showed significantly higher levels of Bifidobacterium and Lactobacillus and lower levels of Enterobacter compared with the control group (*P* < 0·05). Additionally, the observation group had lower levels of SS and NO (*P* < 0·05), indicating improved brain–gut communication. Clinical efficacy was significantly higher in the observation group (*P* < 0·05), with improved serum trace element levels (*P* < 0·05 for Ca, Zn and Fe). Furthermore, the recurrence rate 6 months post-treatment was significantly lower in the observation group compared with the control group (*P* < 0·05). Probiotic supplementation in children with AN effectively modulates intestinal flora, improves brain–gut peptide levels and enhances clinical outcomes.

Anorexia nervosa (AN) is a severe psychiatric disorder characterised by extreme dietary restriction, leading to significant weight loss and malnutrition. This condition is particularly prevalent among children and adolescents, with complex aetiologies involving genetic, psychological and social factors^(1,2)^. AN is also accompanied by an intense fear of obesity and a distorted body image. If not properly treated, it can result in arrhythmias, severe malnutrition, electrolyte and metabolic imbalances, multiple organ failure and even death. AN not only impacts the physical health of affected children but also has profound negative effects on their psychological and social functioning, posing a serious risk to life in severe cases^(3,4)^. Potential treatments include psychotropic medications and novel neuroendocrine agents that can alleviate complications and regulate body weight. However, due to the low tolerance of adolescents to medication, there are significant concerns regarding the safety of using psychotropic drugs, neuroendocrine factors and novel neurotransmitter-targeted therapies. Currently, the treatment of AN faces numerous challenges, with traditional approaches offering limited effectiveness, highlighting the urgent need to explore new treatment strategies to improve patient outcomes^(5,6)^. The limitations of existing treatments underscore the urgent need to explore novel therapeutic strategies to improve outcomes in paediatric patients with AN.

Probiotics, defined as live micro-organisms that provide health benefits to the host when administered in adequate amounts, have gained increasing attention in recent years due to their role in regulating intestinal flora and promoting gut health^(7)^. Probiotics are typically classified into three main groups: lactic acid bacteria, bifidobacteria and gram-positive cocci. Research suggests that probiotics may restore the balance of intestinal flora through mechanisms such as competitive exclusion of pathogens, modulation of immune responses and improvement of intestinal barrier function^(8)^. Furthermore, probiotics have demonstrated the ability to influence brain function and behaviour through the gut–brain axis, making them a promising avenue for addressing psychiatric and digestive disorders^(9)^.

Studies have shown that probiotics can alleviate anxiety and depressive symptoms in animal models. For instance, the combination of Lactobacillus helveticus and Bifidobacterium longum has been reported to reduce stress-induced changes in gut permeability and improve mood-related behaviours^(10)^. Clinical trials have further demonstrated that probiotics may benefit psychological health in humans. A randomised, double-blind study revealed significant improvements in anxiety and depression scores among participants who consumed probiotics for 30 d^(11)^. However, research on the application of probiotics in treating AN remains limited, particularly regarding their impact on gut microbiota and brain–gut peptide levels.

This study aims to address these gaps by conducting a randomised controlled trial involving 100 paediatric patients with AN. We investigate the effects of probiotic oral therapy on intestinal flora balance, brain–gut peptide levels and clinical outcomes. The findings are expected to provide new theoretical insights and clinical references for the comprehensive treatment of AN, further expanding the potential applications of probiotics in this field.

## Materials and methods

### Research data

The study was approved by the Eighth Hospital of Shijiazhuang. Recruitment was carried out through clinical medical records, hospital outpatient visits and physician referrals. Paediatric patients with AN admitted to Xingtang County People’s Hospital between January 2023 and June 2024 were included in the study. Patients who met the inclusion criteria were invited to participate, and informed consent was waived by our Institutional Review Board because of the retrospective nature of our study. A total of 100 cases were ultimately included and divided into two groups according to the treatment method: fifty cases in the control group and fifty cases in the observation group.

### Inclusion criteria

Inclusion criteria: Patients meeting the diagnostic criteria for AN as outlined in the ‘Chinese Classification and Diagnosis of Mental Disorders, 3rd Edition (CCMD-3)’; aged 14 years or younger; with normal intelligence and language communication abilities; and whose guardians had been informed, consented and signed the informed consent form.

Exclusion criteria: Children with organic diseases of the liver, biliary tract, pancreas, gastrointestinal tract or other organ systems; children with anorexia due to endocrine abnormalities or severe micronutrient deficiencies; and children who had received antidepressant, antibiotic or gastrointestinal motility drug treatments in the past 4 weeks^(7,8)^.

### Methods

Both groups received dietary and exercise guidance during the treatment period. The children’s families were instructed on appropriate feeding practices and how to correct picky eating behaviours. In addition to this, the control group was administered zinc gluconate oral solution (Huzhou Huaren Laotongjun Pharmaceutical Co., Ltd, production batch number 20170915, specification 10 ml per vial) orally. The dosage was 10 ml once daily for children aged 1–8 years, and 10 ml twice daily after meals for children aged 9 years and older. The observation group was additionally treated with a quadruple probiotic tablet (Hangzhou Yuanda Biopharmaceutical Co., Ltd, National Medicine Standard Approval Number S200060010, specification 0·5 g per tablet), with one tablet taken orally twice daily. Both groups followed an 8-week treatment course.

### Observational indicators

Intestinal flora: Wet stool samples (3 g) were collected before and after treatment. Samples were placed in tubes containing DNA stabilisers, rapidly frozen on dry ice and stored at −80°C. Total bacterial DNA was extracted using an ELISA-based method, and levels of Bifidobacterium, Lactobacillus and Enterobacter were measured. The instruments were supplied by Beijing EnoGene Biotechnology Co., Ltd.

Brain–gut peptides: Approximately 5 mL of fasting venous blood was collected before and after treatment. Blood samples were centrifuged at 3000 rpm for 5 min to isolate serum, which was stored at −80°C. Serum levels of somatostatin (SS) and nitric oxide (NO) were measured using a RIA. The assay kits were provided by Beijing Pupos Biotechnology Co., Ltd.

Clinical efficacy: Before and after treatment, the symptoms of AN (appetite, food intake and eating time) were semi-quantitatively scored and categorised into four levels: cured (restoration to pre-illness normal food intake or normal intake for children of the same age, weight gain ≥ 0·5 kg, no recurrence within 1 month after stopping medication), markedly effective (restoration to 2/3 of pre-illness normal food intake or normal intake for children of the same age, weight gain 0·25–0·50 kg, AN symptom score reduction ≥ 75 %), effective (restoration to 1/2 of normal food intake or normal intake for children of the same age, weight gain 0·10–0·25 kg, AN symptom score reduction ≥ 30 %) and ineffective (no significant improvement in appetite after treatment, or even worsening, weight gain < 0·1 kg or continued reduction, AN symptom score reduction < 30 % or increase). The total effective rate was calculated.

Trace elements: After treatment, 5 ml of fasting venous blood was collected from the children in the morning, centrifuged at 2500 rpm for 5 min to separate the serum, and the levels of Ca, Zn and Fe in the serum were measured using a fully automated biochemical analyser (Empower Medical Technology Co., Ltd).

Prognosis: The recurrence rate 6 months after treatment was compared between the two groups. Recurrence was defined as a decrease in appetite after it had previously improved.

Adverse reactions (nausea, vomiting, rash and constipation) during treatment in both groups were recorded.

### Data analysis

The data visualisation was performed using GraphPad Prism 9, and statistical analysis was conducted using SPSS 26.0 software. Categorical data were compared using the χ^2^ test, while continuous data were analysed using independent-sample tests. Paired continuous data were analysed using paired sample tests. For data not following a normal distribution, the results were described using the median and interquartile range and analysed using non-parametric rank-sum tests (Mann–Whitney *U* test). A *P* < 0·05 was considered statistically significant.

## Results

### General information

In the control group, there were fifty children: twenty-two males and twenty-eight females; age ranged from 2 to 12 years, with a mean of 6·91 (sd 1·83) years; the course of the disease ranged from 3 to 13 months, with a mean of 8·03 (sd 2·14) months. In the observation group, there were fifty children: twenty-three males and twenty-seven females; age ranged from 2 to 14 years, with a mean of 7·23 (sd 1·94) years; the course of the disease ranged from 3 to 12 months, with a mean of 7·98 (sd 2·06) months. There were no statistically significant differences in the general information between the two groups, indicating comparability (*P* > 0·05), as shown in Table 1.

**Table 1.** Comparison of general information between the two groups of children (x̄ ± s)

		Control group	Observation group	*t*	*P*
Number of cases	–	50	50		
Sex	Male	22	23		
	Female	28	27		
Age (years)	–	2–12	2–14	–	–
	x̄	6·91	7·23	0·848	0·398
	s	1·83	1·94		
Duration of illness	–	3–13	3–12		
	x̄	8·03	7·98	0·119	0·906
	s	2·14	2·06		

### Intestinal flora

**Figure 1.**
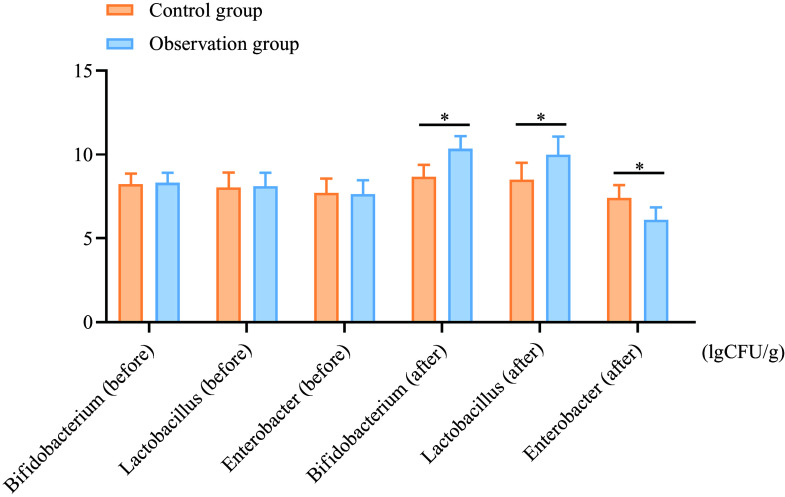
Comparison of intestinal flora counts between the two groups before and after treatment. A: Bifidobacterium; B: Lactobacillus; C: Enterobacter. *P < 0·05.

After treatment, the levels of Bifidobacterium in the observation group increased from 8·31 (sd 0·59) to 10·35 (sd 0·74) (a percentage increase of 24·55 %), while the control group showed an increase from 8·24 (sd 0·62) to 8·65 (sd 0·73) (percentage increase of 4·98 %). Similar trends were observed for Lactobacillus levels, where the observation group showed an increase from 8·11 (sd 0·80) to 9·99 (sd 1·08) (a percentage increase of 23·17 %), compared with the control group, which increased from 8·04 (sd 0·88) to 8·49 (sd 1·01) (percentage increase of 5·59 %). The levels of enterobacteria decreased by 19·79 % in the observation group, compared with a 4·04 % decrease in the control group. These reductions and increases were more significant in the observation group compared with the control group (*P* < 0·05), as shown in Figure 1.

### Brain–gut peptides

**Figure 2.**
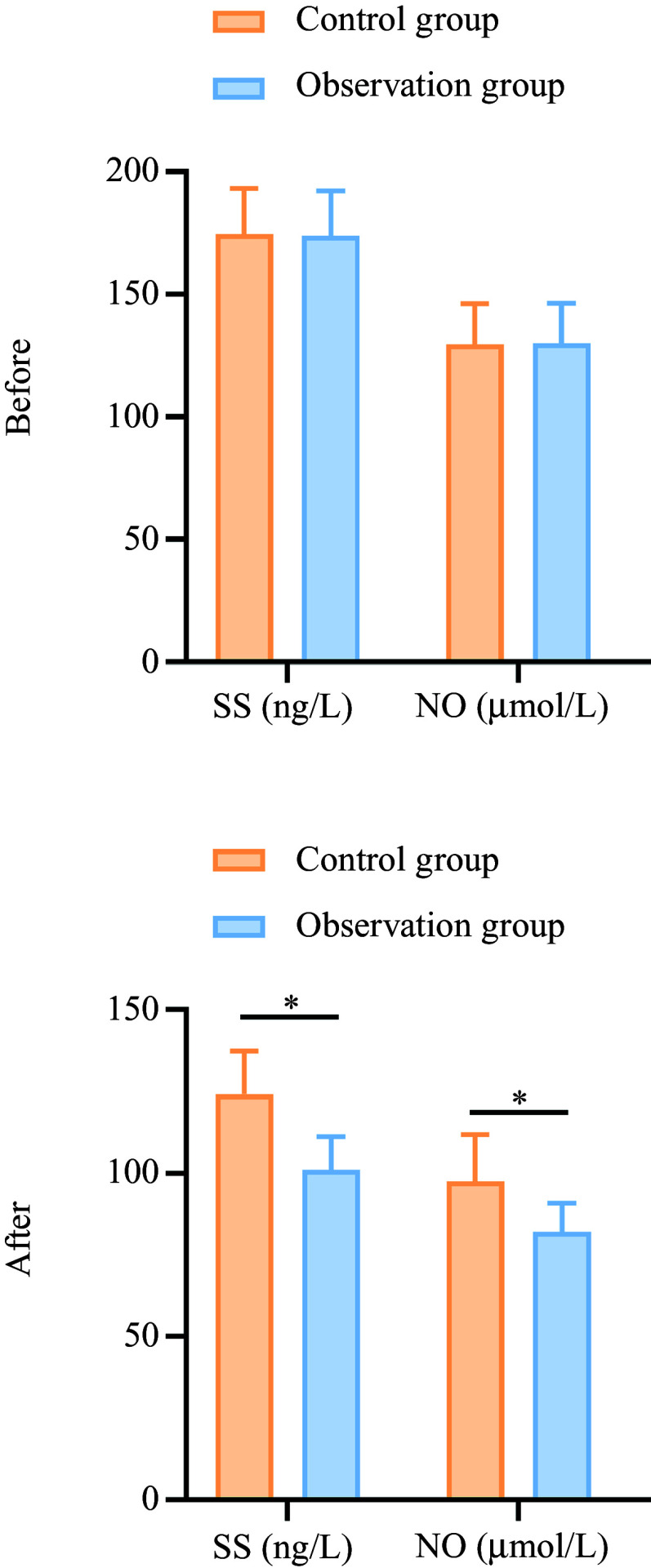
Comparison of brain–gut peptide levels between the two groups before and after treatment. A: SS; B: NO. *P < 0·05. SS, somatostatin; NO, nitric oxide.

The SS levels in the observation group decreased from 173·98 (sd 18·23) to 101·03 (sd 10·17) (a percentage decrease of 41·91 %), while the control group showed a decrease from 174·65 (sd 18·56) to 124·18 (sd 13·26) (percentage decrease of 28·93 %). The NO levels in the observation group decreased from 129·96 (sd 16·45) to 82·03 (sd 8·84) (a percentage decrease of 36·92 %), while the control group showed a decrease from 129·45 (sd 16·87) to 97·56 (sd 14·25) (percentage decrease of 24·65 %). These reductions were more significant in the observation group compared with the control group (*P* < 0·05), as shown in Figure 2.

### Clinical efficacy

**Figure 3.**
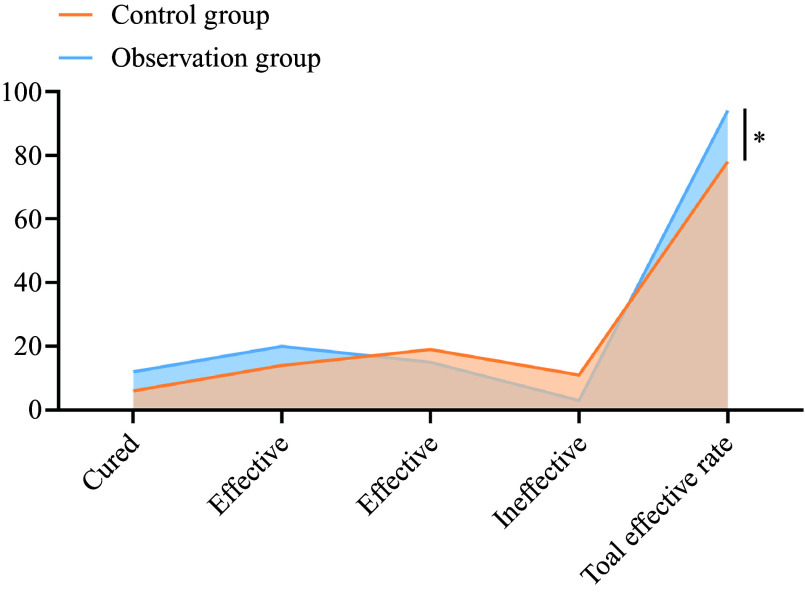
Comparison of clinical efficacy between the two groups. *P < 0·05.

In the control group, six cases were cured, fourteen were effective, nineteen were moderately effective and eleven were ineffective. The total effective rate in the control group was 78 %. In the observation group, twelve cases were cured, twenty were effective, fifteen were moderately effective and three were ineffective. The total effective rate in the observation group was 94 %. The total effective rate in the observation group was 94 % and was significantly higher than that in the control group (78·00 %) (*P* < 0·05), as shown in Figure 3.

### Trace elements

**Figure 4.**
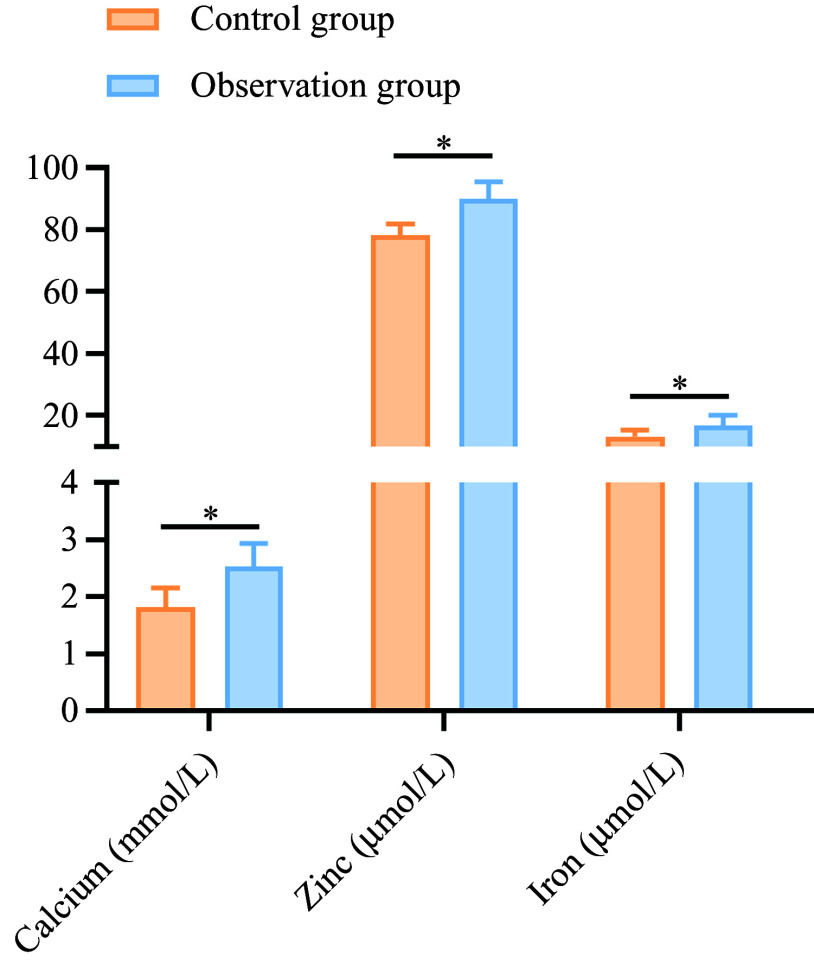
Comparison of trace element levels between the two groups after treatment. *P < 0·05.

After treatment, the levels of serum Ca, Zn and Fe (2·53 (sd 0·41), 90·03 (sd 5·56) and 16·84 (sd 3·11)) in the observation group were higher than those in the control group (1·81 (sd 0·35), 78·22 (sd 3·52) and 13·22 (sd 2·18)) (*P* < 0·05), as shown in Figure 4.

### Prognosis

**Figure 5.**
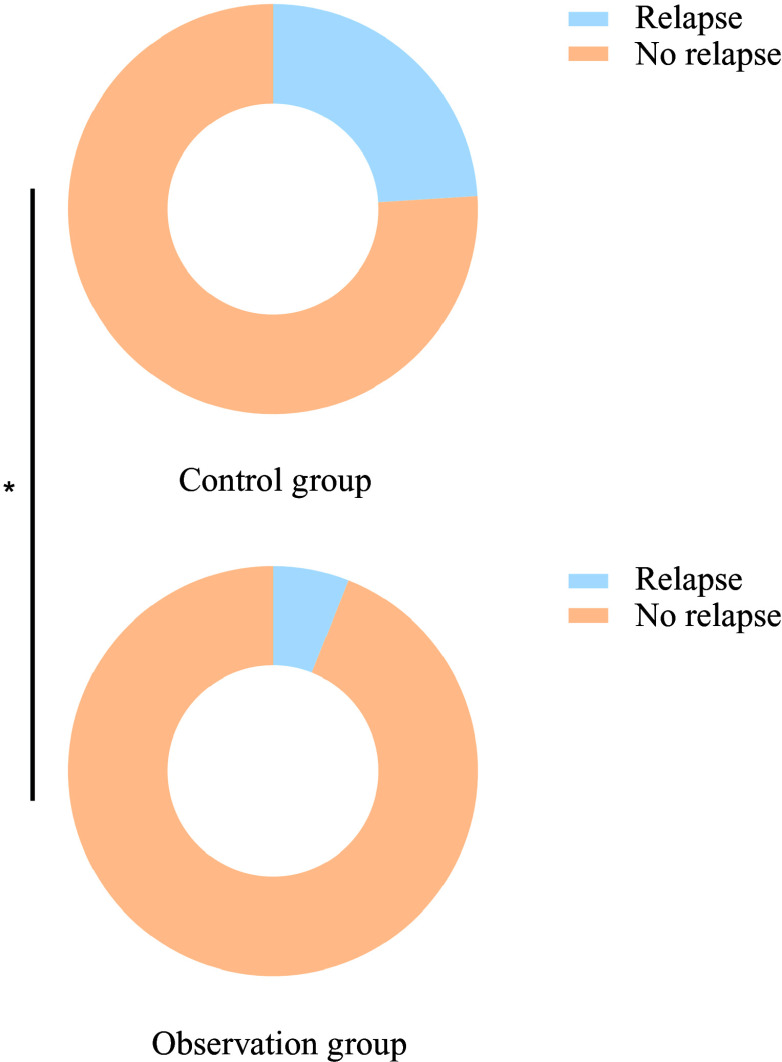
Comparison of recurrence rates between the two groups 6 months after treatment.

Six months after treatment, the recurrence rate in the observation group (6·00 %) was significantly lower than that in the control group (24·00 %) (*P* < 0·05), as shown in Figure 5. No adverse reactions occurred during the treatment period.

## Discussion

The clinical manifestations of paediatric anorexia are primarily characterised by reduced appetite and food intake, which can easily lead to anaemia, malnutrition and other complications. Factors influencing this condition include alterations in intestinal microecology, insufficient secretion of digestive enzymes, micronutrient deficiencies and gastrointestinal dysfunction. Common clinical treatments involve probiotics, prokinetic agents and micronutrient supplementation^(12,13)^.

Zinc gluconate is a micronutrient supplement that can quickly restore taste sensitivity in children, ensuring adequate Zn supply to various enzymes in the body to enhance their activity, thereby improving digestive function and increasing appetite^(14)^. However, using zinc gluconate oral solution alone cannot effectively correct the disrupted microecological environment in children with anorexia, resulting in suboptimal outcomes. The oral administration of Bifidobacterium quadruple viable tablets directly supplements intestinal probiotics, restoring the balance of microbial populations and the host–microbe interaction in the gut, ensuring systemic stability.

Bifidobacteria constitute the majority of dominant intestinal bacteria and play a critical role. Metabolic byproducts such as lactic acid and acetic acid lower intestinal pH and redox potential, promoting the absorption of Ca, Fe and vitamin D, improving the utilisation rates of Ca, phosphorus and Fe, and facilitating intestinal motility and gastric emptying. This reduces the retention time of food in the gastrointestinal tract, inducing hunger and increasing appetite while lowering constipation rates. Additionally, the colonisation of bifidobacteria in the intestine enhances gut immunity by stimulating IgA plasma cell production and activating phagocytic activity, thereby boosting overall immunity and aiding recovery^(15,16)^. Bacillus cereus, which is not a member of normal gut microbiota, consumes oxygen in the gut during its transient colonisation (lasting approximately 48 h before being excreted in feces), creating an anaerobic environment for the colonisation of the other three probiotic strains.

The findings of this study indicate that probiotics play a crucial role in improving symptoms of paediatric AN by restoring gut microbiota balance and regulating brain–gut peptide levels. The gut–brain axis, an important bidirectional communication pathway, has been increasingly recognised as central to the pathophysiology of AN. Probiotics, as key modulators of gut microbiota, offer a novel therapeutic approach addressing both the biological mechanisms and clinical manifestations of the condition.

There is a close association between anorexia in children and reduced levels of micronutrients, including Ca, Zn and Fe. Ca maintains acid–base balance, regulates enzyme activity and suppresses inflammation, while Zn is a crucial component of gustin, essential for taste sensitivity. Fe, a key element of many enzymes, prevents Fe deficiency anaemia, anorexia and digestive dysfunction^(17,18)^. This study demonstrated that after 4 weeks of treatment, serum levels of Ca, Zn and Fe increased significantly in both groups, with levels in the observation group being markedly higher than those in the control group (*P* < 0·05). These results suggest that the combination of Bifidobacterium quadruple viable tablets and zinc gluconate can enhance the levels of these essential micronutrients in school-aged children with anorexia.

Recent literature^(19)^ has reported that intestinal dysbiosis plays a major role in the pathogenesis of paediatric anorexia. A decrease in beneficial bacteria and overgrowth of pathogenic bacteria impair digestive and absorptive capacity while exacerbating micronutrient deficiencies. The results of this study showed that after 4 weeks of treatment, the levels of bifidobacteria and lactobacilli increased significantly, while Enterobacter levels decreased in the observation group. Compared with the control group, the bifidobacteria and lactobacilli levels were significantly higher, and Enterobacter levels were significantly lower in the observation group (*P* < 0·05). This suggests that the combination therapy of Bifidobacterium quadruple viable tablets and zinc gluconate demonstrated superior clinical efficacy compared with zinc gluconate alone. This synergistic effect may be attributed to probiotics enhancing the bioavailability of micronutrients. SCFA, as key metabolic byproducts, not only increase Ca solubility and absorption in the colon but also repair the intestinal mucosa, improving Zn and Fe uptake. Given the common micronutrient deficiencies in AN, which exacerbate the condition, the combination therapy effectively addresses both microbial imbalances and nutritional deficiencies, achieving more comprehensive disease management^(20,21)^.

The observed clinical improvement and reduced recurrence rate during follow-up further support these findings. The lower recurrence rate in the probiotic group suggests that probiotics can provide sustained therapeutic benefits by targeting the underlying mechanisms of gut–brain axis dysfunction and nutritional deficiencies. This aligns with emerging evidence indicating that long-term modulation of gut microbiota plays a critical role in preventing disease relapse and promoting recovery^(22,23)^.

Beyond their effects on the gut microbiota, probiotics also regulate brain–gut peptides closely associated with appetite and gastrointestinal function, such as SS and NO. This study found that SS levels in the observation group were significantly lower than those in the control group after treatment (*P* < 0·05). SS, as an inhibitory neuropeptide, suppresses appetite by inhibiting hunger hormone secretion^(24)^. The regulation of SS secretion by probiotics suggests their potential to modulate appetite-related pathways indirectly through gut microbiota restoration. Similarly, the significantly lower NO levels observed in the observation group (P < 0·05) indicate that probiotics mitigate oxidative stress and intestinal inflammation, further promoting gastrointestinal recovery and appetite enhancement^(25,26)^.

Despite the positive results of this study, there are some limitations. First, the relatively small sample size may affect the generalisability of the results. Second, the study was conducted at a single centre, and the findings may not be directly applicable to other regions or populations. Future studies should investigate the comparative efficacy of various probiotic strains and combinations in treating paediatric anorexia, particularly in addressing specific symptoms such as gastrointestinal dysfunction and micronutrient malabsorption. Additionally, multicentre, large-scale randomised controlled trials are necessary to validate these findings. Long-term follow-up studies are also crucial for evaluating the sustainability of probiotic therapy in preventing relapse and supporting recovery.

### Conclusion

This study demonstrates that the combination of probiotics and zinc gluconate effectively improves the symptoms of paediatric anorexia by restoring gut microbiota balance, regulating brain–gut peptides and enhancing micronutrient absorption. These findings highlight the potential of probiotics as an adjunctive therapy for childhood anorexia and underscore the importance of addressing both microbial and nutritional imbalances.

The findings provide a strong rationale for incorporating probiotics into the clinical management of paediatric anorexia, alongside micronutrient supplementation. Future research should focus on optimising probiotic formulations, understanding the mechanisms of gut–brain interactions and evaluating long-term outcomes in diverse populations
